# Has COVID-19 changed the approach to HIV diagnosis? A multicentric Italian experience

**DOI:** 10.1097/MD.0000000000027418

**Published:** 2021-10-15

**Authors:** Maria Mazzitelli, Arturo Ciccullo, Gianmaria Baldin, Roberto Cauda, Stefano Rusconi, Andrea Giacomelli, Letizia Oreni, Vanni Borghi, Cristina Mussini, Giovanni Guaraldi, Gaetana Sterrantino, Filippo Lagi, Bianca Candelaresi, Oscar Cirioni, Andrea De Vito, Barbara Rossetti, Carlo Torti, Simona Di Giambenedetto

**Affiliations:** a Infectious and Tropical Diseases Unit, Department of Medical and Surgical Sciences, “Magna Graecia” University of Catanzaro, Catanzaro, Italy; b Gemelli Molise Hospital, Campobasso, Italy; c Mater Olbia Hospital, Olbia, Italy; d Catholic University of the Sacred Heart, Rome, Italy; e Fondazione Policlinico Universitario “Agostino Gemelli” IRCCS, Rome, Italy; f III Infectious Diseases Unit, ASST FBF-Sacco, Milan, Italy; g UOC Malattie Infettive, Ospedale di Legnano, ASST Ovest Milanese, Italy; h DIBIC Luigi Sacco, Università degli Studi di Milano, Milan, Italy; i Clinica Malattia Infettive, Università di Modena e Reggio Emilia; j Dipartimento di Medicina clinica e Sperimentale presso l’Università degli studi di Firenze; k Clinica Malattie Infettive, Azienda Ospedaliero-Universitaria Ospedali Riuniti di Ancona, Ancona, Italy; l Unit of Infectious Diseases, Department of Medical, Surgical and Experimental Sciences, University of Sassari, Sassari, Italy; m UOC Malattie Infettive e Tropicali, Azienda Ospedaliero-Universitaria Senese, Siena, Italy.

**Keywords:** AIDS, COVID-19, HIV, late presentation, lockdown

## Abstract

The occurrence of COVID-19 pandemic had a significant negative effect on health care systems over the last year. Health care providers were forced to focus mainly on COVID-19 patients, neglecting in many cases equally important diseases, both acute and chronic. Therefore, also screening and diagnostic strategies for HIV could have been significantly impaired.

This retrospective, multicenter, observational study aimed at assessing the number and characteristics of new HIV/AIDS diagnoses during COVID-19 pandemic in Italy and compared characteristics of people living with HIV at diagnosis between pre- and post-COVID-19 era (2019 vs 2020).

Our results showed a significant reduction of HIV diagnoses during pandemic. By contrast, people living with HIV during pandemic were older and were diagnosed in earlier stage of disease (considering CD4+ T cell count) compared to those who were diagnosed the year before. Moreover, there was a significant decrease of new HIV diagnoses among men who have sex with men, probably for the impact of social distancing and restriction applied by the Italian Government. Late presentation incidence, if numbers in 2020 were lower than those in 2019, is still an issue.

Routinely performing HIV testing in patients with suspected SARS-CoV-2 infection is identifying and linking to care underdiagnosed people living with HIV earlier. Thus, combined tests (HIV and SARS-CoV-2) should be implemented in patients with SARS-CoV-2 symptoms overlapping HIV's ones. Lastly, our results lastly showed how urgent implementation of a national policy for HIV screening is necessary.

## 1 Introduction

From the latest 2019, a new coronavirus, causing a severe form of pneumonia, was identified, and subsequently named severe acute respiratory syndrome Coronavirus-2 (SARS-CoV-2). It causes the Coronavirus Disease 2019 (COVID-19), responsible of a pandemic status that is ongoing from the march 2020. COVID-19 pandemic is still forcing a radical reshape of the health care system, impairing its ability to assist patients with chronic diseases. Indeed, HIV care was importantly altered in several aspects. Routine clinical checks for subjects already in care in HIV clinics were reduced or replaced by telemedicine, many countries were at risk of stocking-out antiretrovirals and, importantly, clinical experiences demonstrated significant reductions of screening and prevention strategies for HIV.^[1]^

Since severe acute respiratory syndrome Coronavirus-2 (SARS-CoV-2) and HIV infections have several symptoms and signs in common, a confounding effect may lead to underdiagnosis of HIV conditions, such as acute infections or even AIDS-defining illnesses. Moreover, it is still object of debate whether HIV increase the risk of acquiring or developing a more severe COVID-19.

Objectives of this study were: to evaluate the number and characteristics of new HIV/AIDS diagnoses during COVID-19 pandemic in Italy and to compare characteristics of people living with HIV (PLWH) at diagnosis between pre- and post-COVID-19 era.

## 2 Methods

We conducted a retrospective multicenter descriptive cross-sectional study in centers participating to the ODOACRE Cohort study.^[2]^

We included in our analysis all people older than 18 years of age, who got a confirmed HIV test as positive in the following 2 time frames: from March 1^st^ to 31^st^ July 2019 (pre COVID-19 era) and from March 1^st^ to 31^st^ July 2020 (COVID-19 era). Only patients who presented for clinical care in the 8 centers participating to the study were enrolled. Data from 18 Italian clinical centers were included. Clinical, laboratory results, and demographics (age, sex, country of origin) were collected from medical records. Descriptive analysis by using parametric (*χ*^2^ test or Fisher exact test) and nonparametric (Mann–Whitney *U* test) tests was conducted as appropriate. *P* values <.05 were considered as statistically significant. Shapiro-Wilk analysis was used to test the normality of distribution.

### 2.1 Ethical review

ODOACRE study protocol received approval from ethics committees of every participating Center. Patients recruited in ODOACRE Cohort were asked to sign an informed consent.

## 3 Results

A total of 131 patients were included in this analysis: 77 (58.8%) of the “pre COVID-19” group and 54 (41.2%) of the “COVID-19” group. Full characteristics of patients who were included are depicted in Table 1. Patients’ median age was 36 years (interquartile range [IQR]: 28–48), median CD4+ T-cell count at HIV diagnosis was 227 cells/mm^3^ (IQR: 104–451), whereas median HIV-RNA was 4.98 log_10_ copies/mL (IQR: 4.40–5.47). Male sex was predominant in the 2 periods. No differences were found between the 2 period as for nationalities and risk factors for HIV acquisition, except for the prevalence of new diagnoses among men who have sex with men (MSM) (47/77 in pre-COVID-19 era vs 19/54 during COVID-19 era, *P* = .002). We observed significant differences between pre-COVID-19 and COVID-era regarding CD4+ T-cell count at diagnosis (median: 205 vs 305 cells/mm^3^, *P* = .026) and zenith HIV-RNA (median: 5.21 log_10_ vs 4.46 log_10_ copies/mL, *P* < .001). As for antiretroviral regimens, the majority of patients started a 3-drug regimen with 2 nucleos(t)ide reverse-transcriptase inhibitors plus integrase inhibitors (96, 73.3%). Regarding AIDS-defining illnesses, 24 (31.2%) PLWH in pre-COVID and 6 (11.1%) in COVID era were diagnosed with such illnesses and therefore had a CDC stage C disease (*P* = .011). Fifty-one (66.2%) subjects during pre-COVID-19 era and 31 (57.4%) subjects during COVID-19 era were late presenters (*P* = .004).^[3]^ In the COVID-era, 17 (31.5%) subjects at the time of HIV diagnosis showed at least 1 symptom compatible both with COVID-19 and HIV infection. During our study period, 22 (40.7%) subjects were tested for SARS-CoV-2 and 2 (3.7%) were positive. No differences in percentages of symptoms were found at presentation between 2019 and 2020.

**Table 1:** Cohort description.

Characteristics	Pre-COVID-19 era, N = 77	COVID-19 era, N = 54	*P*
Age, median (IQR)	34.0 (27.0–46.4)	39.9 (28.9–49.9)	.088
Sex n (%)			.267
Male	61 (79.2)	36 (66.7)	
Female	12 (15.6)	14 (25.9)	
Transgender	4 (5.2)	4 (7.4)	
Country, n (%)			.167
Italy	44 (57.1)	31 (57.4)	
Africa	12 (15.6)	2 (3.7)	
Asia	3 (3.9)	4 (7.4)	
Eastern Europe	4 (5.2)	6 (11.1)	
South America	14 (18.2)	11 (20.4)	
Risk factor, n (%)—∗each patient may have more than one			
Men who have sex with men	47 (61.0)	19 (35.2)	.002
Heterosexual	31 (40.3)	31 (57.4)	.077
Intravenous drug use	0 (0)	2 (3.7)	.168
Other	2 (2.6)	4 (7.4)	.674
AIDS-defining illness	24 (31.2)	6 (11.3)	.011
Late presentation	49 (63.6)	31 (57.4)	.004
Screening for SARS-CoV-2		22 (40.7)	—
Positive result		2 (3.7)	—
Nadir CD4+ cell count, median (IQR)	205 (59–432)	305 (157–553)	.026
Zenith HIV-RNA (log10 copies/mL), median (IQR)	5.22 (4.78–5.78)	4.46 (4.09–4.92)	<.001
Symptoms at presentation			
Cough	7 (9.1)	6 (11.3)	1
Fever	9 (11.7)	13 (24.1)	.457
Shortness of breath	2 (2.6)	6 (11.3)	.145
Diarrhea	3 (3.9)	3 (5.6)	1
Weight loss	5 (6.5)	11 (20.4)	.060
Rash	2 (2.6)	2 (3.7)	1
Sweats	3 (3.9)	4 (7.4)	.705
Headache	1 (1.4)	1 (1.9)	1
Loss of consciousness	0	1 (1.9)	.481
Asthenia	3 (3.9)	3 (5.6)	1
Other	5 (6.5)	2 (3.7)	.194
First-line antiretrovirals			.712
2NRTI + INI	56 (72.7)	40 (74.1)	
2NRTI + PI	9 (11.7)	3 (5.6)	
2NRTI + NNRTI	6 (7.8)	5 (9.2)	
DTG + 3TC	3 (3.9)	4 (7.4)	
Other/none	3 (3.9)	2 (3.7)	
Comorbidities			
Diabetes	1 (1.4)	2 (3.7)	.386
Hypertension	6 (8.2)	6 (11.3)	.558
Ischemic heart disease	0	0	N/A
Obesity	1 (1.4)	0	.454
Osteoporosis	0	2 (3.7)	.499
Malignancy	5 (6.5)	2 (3.7)	.698
Dyslipidemia	1 (1.4)	4 (7.4)	.161
Chronic kidney disease	0	1 (1.9)	.421
Other	7 (9.1)	0	N/A

## 4 Discussion

The number of new HIV diagnoses in our cohort during the pandemic is still worrying, even if lower compared to the pre-COVID era. This number could have several explanations. A first explanation could be poor implementation of testing strategies and lack of access to hospital for fear of SARS-COV-2 or logistical constraints of the overstressed public health system.^[4]^ Second, travel reduction and lockdown restrictions applied by the Government to contain the spreading of SARS-CoV-2 infection could have had an impact on risky behaviors, especially among MSM.^[5]^ Also, due to restrictions, most Italian migrants moved from their workplace to their place of origin, for work-from-home strategies. This phenomenon could have made the detection of new HIV diagnoses fail either because in their place of origin (usually small cities or villages throughout Italy) testing strategies could be sub-optimally implemented, or simply because diagnosis was made out of the network of our study.^[6]^ Whatever are the reasons, our study has important implications for HIV testing strategies, suggesting that routine HIV testing should be promoted not only in the biggest cities (such as “Fast Track Cities for HIV”), but also in small centers, creating a more capillary screening network. As for risk factors, a statistically significant difference was found in the new diagnoses for MSM, with a significant lower percentage of MSM diagnosed in COVID-era than those diagnosed in pre-COVID era. This result appears in contrast with epidemiological data showing an increasing trend of PLWH among MSM and may be due to the fact this population had fewer opportunities to be tested for fear of contagion or movement to other cities.^[7]^

Our study demonstrated that late presentation of HIV patients still remains significant (57%), although it appears that this percentage is reduced if compared to the pre-COVID era (64%), CD4+ T cell counts were greater and HIV-RNA levels were lower at presentation. It is reasonable to think that the fear to be SARS-CoV-2-infected led people who had symptoms to access earlier to health care points, paying more attention to any kind of symptoms to guarantee a rapid differential diagnosis. This is confirmed by previous results showing, in particular, a significant increase of acute HIV diagnoses due to fear of COVID-19.^[8]^ Notwithstanding the prognostic value of baseline CD4+ T cell count in PLWH decreased over time, severe immunosuppression still remain an important factor for HIV progression and death.^[9]^ Indeed, patients who have a very low CD4 + T cell count when are diagnosed with HIV are more likely to develop AIDS events, even if on stable treatment with antiretrovirals.^[9]^ Percentages of late presentations, independently from the possible effect of COVID-19 on HIV testing strategies, were higher than those previously reported.^[10]^ In 2020 and in 2019, late presentations on the amount of new HIV diagnosis in our study were 63.6% and 57.4%, respectively. These number are slightly higher than those described in a multicenter evaluation previously conducted in Italy where late presenter percentage was 56.9%, even if mortality significantly decreased over time.^[10]^ Paradoxically, with the evolution of HIV testing and implementation of screening campaigns, percentages of late presentation continued to increase in Italy from 1985.^[10]^

The advantage of routinely performing HIV testing in patients with suspected SARS-CoV-2 infection is identifying and linking to care underdiagnosed PLWH earlier. Therefore, combined tests (HIV and SARS-CoV-2) should be implemented in patients with SARS-CoV-2 symptoms overlapping HIV's ones.

At the same time, clinicians should pay attention to cross-reactivity between SARS-CoV-2 tests with HIV chemiluminescent assay. Indeed, recent data showed how several patients can have positivity both at serum HIV screening tests and at nasopharyngeal swabs for SARS-CoV-2. ^[11]^ However, the risk of this cross-reactivity was not present in our study, as only confirmed cases of PLWH were included.

This study presents several limitations, including its cross-sectional nature, limited number of participating centers, and the lack of possibility to establish the actual burden of the new HIV/AIDS diagnoses, including those made in patients not followed in centers not included in the present cohort. Also, proportion of acute HIV infections was not assessed in the present study. Despite limitations, our experience supports three main conclusions. The first one is the fact that pandemic could have significantly impaired HIV diagnoses in our Country, representing a significant public health issue; the second one is that HIV testing should be included in the diagnostic algorithm of patients with suspected SARS-CoV-2 infection; lastly, a national policy for HIV testing and care should be implemented throughout the entire Country.

## Author contributions

MM, CT, AC, SDG, RC were involved in designing the study. MM, AC, SR, AG, LO, VB, CM, GG, GS, FL, BC, GM, BS collected the data. MM and AC analyzed the data. All authors were involved in writing and revising the article.

**Conceptualization:** Maria Mazzitelli, Arturo Ciccullo, Roberto Cauda, Carlo Torti, Simona Di Giambenedetto.

**Data curation:** Maria Mazzitelli, Arturo Ciccullo, Gianmaria Baldin, Stefano Rusconi, Andrea Giacomelli, Letizia Oreni, Vanni Borghi, Cristina Mussini, Giovanni Guaraldi, Gaetana Sterrantino, Filippo Lagi, Bianca Candelaresi, Oscar Cirioni, Andrea De Vito, Barbara Rossetti, Carlo Torti, Simona Di Giambenedetto.

**Formal analysis:** Maria Mazzitelli, Arturo Ciccullo.

**Methodology:** Maria Mazzitelli.

**Validation:** Maria Mazzitelli.

**Writing – original draft:** Maria Mazzitelli, Stefano Rusconi.

**Writing – review & editing:** Maria Mazzitelli, Arturo Ciccullo, Gianmaria Baldin, Roberto Cauda, Stefano Rusconi, Andrea Giacomelli, Letizia Oreni, Vanni Borghi, Cristina Mussini, Giovanni Guaraldi, Gaetana Sterrantino, Filippo Lagi, Bianca Candelaresi, Oscar Cirioni, Andrea De Vito, Barbara Rossetti, Carlo Torti, Simona Di Giambenedetto.
